# 

**Published:** 1866-03

**Authors:** 


### MISSISSIPPI VALLEY DENTAL ASSOCIATION.

### HYMENIAL.

Married on Wednesday the 28th of February ult., Dr. W. .H Shadoan, of Louisville, Ky. to Miss Lucy M. Sabine, of Cincinnati.

This is a subject in which it is rather difficult to introduce much about the teeth, this must be our apology for the brevity of this notice ; it is rather out of our line, at least it is not our forte. Yet we must express our ardent wishes for the perpetuity of the connubial happiness that now pervades the bosoms of the blissful pair.

AMERICAN DENTAL ASSOCIATION—Meets annually (this year at Boston,) *Pres.* C. W. SPALDING, cf St. Louis, *Rec. Sec.* J. TAFT, of Cincinnati, *Cor. Sec.* L. D. SHEPARD, of Salem, Mass.

*List of Societies represented in the American Dental Association.*

ALBANY DENTAL ASSOCIATION—Meets monthly at Albany. *Pres.* ROBERT NELSON *Rec Sec.* W. F. WINNE, *Cor. Sec.* B. WOOD, of Albany.

BUFFALO DENTAL SOCIETY—Meets monthly at Buffalo. *Pres.* R. G. SNOW, *Rec Sec.* GEO. B. SNOW, of Buffalo.

BROOKLYN DENTAL ASSOCIATION—Meets semi-monthly *Pres.* W. C. PARKS, *Rec. Sec.* W. B. HURD, *Cor. Sec.* W. H. ATKINSON, of New York.

CINCINNATI DENTAL SOCIETY—Meets semi-monthly at Cincinnati. *Pres.* C. H. JAMES, *Rec. Sec.* WM. TAFT.

CENTRAL OHIO DENTAL ASSOCIATION—Meets Semi-annually* on the second Tuesday of May and November. *Pres.* Dr. J. ARMSTRONG, Bucyrus, O., *Sec.* A. W. MAXWELL Gallion, O.

CENTRAL STATES DENTAL ASSOCIATION—Meets annually* *Pres.* Dr. W H. MORGAN, *Sec.* W. H. SHADOAN, Louisville, Ky.

CENTRAL NEW YORK DENTAL ASSOCIATION Meets semi-annually. *Pres.* S. B. PALMER, *Rec. Sec.* S. G. MARTIN, *Cor. Sec.* P. HARRIS of Skaneatles.

CHICAGO DENTAL SOCIETY—Meets monthly at Chicago. *Pres.* J. W. ELLIS, *Rec. and Cor. Sec.* E. W. SAWYER of Chicago.

CONNECTICUT VALLEY DENTAL ASSOCIATION—Meets semi-annually. second Tuesday of May and October, *Pres.* J. BEALS. *Rec. Sec.* L. D. SHEPHERD, of Salem, Mass.

HUDSON VALLEY DENTAL ASSOCIATION—meets monthly at Troy, N. Y. *Pres.* H. H. YOUNG, *Rec. Sec.* S. J. ANDRES, *Cor. Sec.* S. P. WELCH, of Troy.

INDIANA STATE DENTAL SOCIETY—Meets annually at Indianapolis on the last Tuesday of June. *Pres.* A. M. MOORE, *Rec. and Cor. Sec* JOS. RICHARDSON, of Terre Haute, Ind

IOWA STATE DENTAL SOCIETY—Meets semi-annually Jan. and July. *Pres.* L. C. INGERSOLL, *Rec. Sec.* H. S. CHASE.

LEBANON VALLEY DENTAL ASSOCIATION—*Prest.* W. K. BRENIZER.

MAD RIVER VALLEY DENTAL SOCIETY—Meets quarterly. Next meeting in Dayton, on the first Tuesday in June next. *Prest.*, M. M. OLDHAM, *Sect.*, J. BLAKE.

MICHIGAN DENTAL SOCIETY—Meets annually fourth Tuesday of Jan. at Detroit- *Pres.* J. W. FIELD, *Rec. and Cor. Sec.* G. W. STONE, Albion.

MISSISSIPPI VALLEY DENTAL ASSOCIATION—Meets annually in February at Cincinnati, *Pres.* G W KEELY. *Rec. Sec.* WM. TAFT, *Cor Sec.* H. A. SMITH, of Cin.

MISSOURI DENTAL ASSOCIATION—Meets on the first Tuesday of June, 1866, in St' Louis. *Pres.* Dr. H. J. MCKELLOPS. St. Louis, *Sec* H. JUDD, St. Louis.

MERRIMAC VALLEY DENTAL SOCIETY—Meets semi annually* first Tuesday of Oct. and May. *Pres.* A. LAWRENCE, *Rec. Sec.* G. A. GERRY, of Lowell.

NEW YORK SOCIETY OF DENTAL SURGEONS—Meets semi-monthly in New York, *Pres.* W. H. ALLEN, *Rec. Sec.* C. D. ALLEN, *Cor. Sec.* C. P. FITCH, of New York.

NEW HAVEN DENTAL SOCIETY—Meets on the first Wednesday of each month at New Haven. *Pres.* J. T. METCALF, *Rec. and Cor. Sec.* C. L. SMITH, of New Haven.

NEW LONDON COUNTY DENTAL SOCIETY—Meets monthly.* *Pres.* R. W. BROWNE *Rec. Sec.* WM. ALLENDER.

NORTHERN OHIO DENTAL ASSOCIATION—Meets Semi-annually, (first Tuesday of May and October ) in Cleveland. *Pres.* B. STRICKLAND, *Rec. Sec.* L. BUFFETT, *Cor. Sec* B. F. ROBINSON, Cleveland.

OHIO DENTAL COLLEGE ASSOCIATION—Meets annually in February at Cincinnati. *Pres.* J. H. MCKELLOPS, *Rec. Sec.* J. TAFT, of Cincinnati.

ODONTOGRAPHIC SOCIETY OF PENNSYLVANIA—Meets monthly in Philadelphia. *Pres.* JAS. N. HARRIS, *Rec. Sec.* R. J. HORNER, *Cor. Sec.* J. H. MCQUILLEN, of Phila.

PENNSYLVANIA ASSOCIATION OF DENTAL SURGEONS—Meets monthly in Phila *Pres.* W. W. FOUCHE, *Rec. Sec* JAMES TRUMAN, *Cor. Sec.* G. W. ELLIS, of Philadelphia

PITTSBURG DENTAL ASSOCIATION—Meets monthly, (second Tuesday, in Pittsburg. *Pres*. M. E. GILLESPIE, *Rec. Sec*. J. D. WHITE.

PENINSULAR DENTAL SOCIETY—Meets quarterly.* *Pres*. SAMUEL MARSHALL, *Rec. Sec*. W. G. A. BONWILL, *Cor. Sec*. S. S. NONES.

WESTERN DENTAL SOCIETY—Meets annually.* *Pres*. T. F. ABELL, *Rec. Sec*. C. W. SPALDING, *Cor. Sec*. E. A BOGUE.

ST. LOUIS DENTAL SOCIETY—Meets monthly at St. Louis.

WESTERN NEW YORK DENTAL ASSOCIATION—Meets semi-annually, *(next meeting at Lockport,) *Pres*. GEO. E. HAYES, *Sec* GEO. B. SNOW.

ST. LOUIS ODONTOLOGICAL SOCIETY—*Pres*. E. HALE, *Rec. Sec*. G. H. SILVERS, *Cor. Sec*. G. G. SAMUEL.

AMERICAN DENTAL CONVENTION—Meets annually first Tuesday of August, (next meeting at New York,) *Pres*. H. E. PEEBLES, *Rec. Sec*. H. A. SMITH. *Cor. Sec*. W. H. ALLEN, of New York.

**Place of meeting fixed by appointment*

### CONTRIBUTORS.

W. H. ATKINSON, M. D., D. D. S.
B. WOOD, M. D.
W. H. SHADOAN.
WM. A. PEASE, D. D. S.
LEON F. HARVEY, M. D.
H. A. SMITH D. D. S.
GEO. B. SNOW, D. D. S.
WM. O. KULP.
A. S. TALBERT, D. D. S.
JOS. RICHARDSON, D. D. S.
A. LAWRENCE.
H. S. CHASE, D. D. S.
R. J. POORE.
GEO. L. PAINE. D. D. S.
A. A. BLOUNT, M. D.
G. A. MILLS.
C. W. SPALDING, D.D.S.
J. DOUGLASS.
J. S. LATIMER, D. D. S.
J. D. ANDERSON, D. D. S,
H. BENEDICT, D. D. S.
J. MANSFIELD, D. D. S.
J. F. JOHNSON, D. D. S.
A. M. LESLIE, D. D. S.
GEO. WATT, D. D. S.
J. TAFT, D. D S.
S. B. PALMER.
F. ABBOTT.
A. BERRY, D. D. S.
A. W. MAXWELL.
H. F. BISHOP.
JOSEPH S. HILDRETH, M..D
GAM'L JACKSON.
H. T. CROSSINGER.
J. CHESEBROUGH, D. D. S.
G. W. REDMAN.

### EXCHANGES,

ST. LOUIS MEDICAL AND SURGICAL JOURNAL,
BOSTON MEDICAL AND SURGICAL JOURNAL,
THE PACIFIC MEDICAL AND SURGICAL JOURNAL,
BUFFALO MEDICAL AND SURGICAL JOURNAL,
OHIO MEDICAL AND SURGICAL JOURNAL,
PHILADELPHIA MEDICAL AND SURGICAL JOURNAL,
WESTERN LANCET AND OBSERVER,
CHICAGO MEDICAL JOURNAL,
DENTAL COSMOS,
L' ART DENTAIRE,
THE LONDON DENTAL REVIEW,
BRAITHWAITE'S RETROSPECT,
SAN FRANCISCO MEDICAL PRESS,
THE DENTAL TIMES,
LADIES' REPOSITORY,
HALL'S JOURNAL OF HEALTH,
DEUTSCHE VIERTELIAHRSSCHRIFT,
DENTAL CIRCULAR AND EXAMINER,
CHICAGO MEDICAL EXAMINER.
THE CINCINNATI JOURNAL OF MEDICINE.
MEDICAL RECORDER.
XENIA T RCHLIGHT.
THE U. S. MEDICAL AND SURGICAL JOURNAL.
THE DENTAL QUARTERLY.

### CONTENTS.

OF THE WEST.

*Issued Monthly, at $3 a Year in Advance.*

DEVOTED TO THE INTERESTS OF THE DENTAL PROFESSION.

EDITED BY J. TAFT

The volume commences in January and closes in December.

The Register being issued monthly is always fresh, therefore indispensable to the practitioner who desires to keep posted up with the new inventions and improvements which are daily developed. Neither expense nor effort will be spared to give value and variety to its contents. Articles will be illustrated with well executed engravings whenever they will add to the interest or assist in explanation.

Its extensive circulation and frequency of issue makes it one of the BEST DENTAL ADVERTISING MEDIUMS in the United States.

RATES OF ADVERTISING.

One page, 1 year, $50. Half page, 1 year, $30. Quarter page, or card, 1 year $20.

All advertising bills payable in advance, or at furthest after the issue of the first number containing the advertisement. All transient advertisements to be paid for in advance.

☞ CONTRIBUTIONS SOLICITED.

Address, J. TAFT, or "DENTAL REGISTER," Cincinnati, Ohio.

Business Communications to

**J. TAFT, Publisher,**
No. 172 Race Street.

